# Identification of a Novel Non-V600E BRAF Mutation in Papillary Thyroid Cancer

**DOI:** 10.1155/2024/6621510

**Published:** 2024-03-19

**Authors:** Marco Capezzone, Maja Rossi, Elisabetta Macerola, Silvia Cantara, Francesco Pepe, Eugenia Maria Morabito, Gilda Dalmazio, Sara Bardi, Agostino Ognibene, Massimo Alessandri, Gabriele Materazzi, Luigi De Napoli, Michele Cirianni, Liborio Torregrossa

**Affiliations:** ^1^UOSD of Endocrinology, Misericordia Hospital, Grosseto 58100, Italy; ^2^UOS Molecular Pathology, Hospital Misericordia, Grosseto 58100, Italy; ^3^Department of Surgical, Medical and Molecular Pathology, University Hospital of Pisa, Pisa 56124, Italy; ^4^Department of Medical, Surgical and Neurological Sciences, University of Siena, Siena 53100, Italy; ^5^Department of Public Health, University of Naples Federico II, Naples, Italy; ^6^Division of Endocrine Surgery, Department of Surgical Pathology, University Hospital of Pisa, Pisa 56124, Italy

## Abstract

Papillary thyroid cancer (PTC) is a common endocrine malignancy, and its incidence is reported to be constantly increasing. BRAF mutation is detected in approximately 44% of PTCs, and the most common BRAF mutation is thymine (T) to adenine (A) missense mutation in nucleotide 1796 (T1796A, V600E). Although BRAFV600E represents 95% of all BRAF mutations, uncommon BRAF mutations have been reported in thyroid carcinomas and represent an alternative mechanism of BRAF activation with unclear clinical significance. We report a novel non-V600E mutation (c.1799_1812delinsAT, p.V600_W604delinsD), identified preoperatively with next-generation sequencing (NGS) on the material obtained with fine-needle aspiration cytology (FNAC) performed on a thyroid nodule cytologically suspicious for malignancy in a 35-year-old male patient. The presence of this new variant of BRAF mutation was subsequently confirmed in the postoperative phase by direct Sanger sequencing. In conclusion, we report a new non-V600E variant previously undetected in papillary thyroid cancer. In addition, this case report shows that the NGS technique on cytological tissue allows to detect the presence of rare mutations, thus increasing the diagnostic specificity of molecular analysis.

## 1. Introduction

The V-Raf murine sarcoma viral oncogene homolog B (*BRAF*) encodes for a serine/threonine protein kinase that participates in the MAPK/ERK signalling pathway, which promotes cell proliferation, differentiation, and apoptosis [1]. Activating point mutations in *BRAF* are recurrent in many cancer types, including papillary thyroid carcinoma (PTC) where the prevalence varies from 23 to 62% [2–4]. Particularly, an exon 15-point mutation consisting of a T to A transversion (c.1799T > A) determines a valine-to-glutamate change in codon 600 (p.V600E), which is located in the kinase domain [5]. Interestingly, the V600E accounts for about 90% of all oncogenic BRAF mutations demonstrating a pivotal role in cancer development [6]. Non-V600E *BRAF* alterations are less common in PTC. The few reports investigating the clinical role of non-V600E *BRAF* mutations in thyroid cancer highlight that these alterations are linked with follicular architecture tumors (mainly encapsulated follicular subtypes of PTC) showing a better clinical outcome compared to *BRAF* p.V600E thyroid tumors [7, 8]. However, rare non-V600E *BRAF* mutations have been reported in thyroid tumors with aggressive clinical course [9].

Here, we report on a PTC patient carrying a novel non-V600E *BRAF* mutation.

## 2. Case Presentation

A 35-year-old male with no family history of benign or malignant thyroid neoplasms, and without exposure to radiation, presented with a 40 mm left-sided thyroid nodule seen on neck ultrasonography (US) during an annual checkup. Thyroid functional tests showed a serum TSH level of 1.5 mIU/L (reference range: 0.2–4.0). On US-guided fine-needle aspiration cytology (FNAC), the nodule was diagnosed as suspicious for malignancy (TIR 4 lesion according to the Italian Consensus Guidelines for the Classification and Reporting of Thyroid Nodules) [10]. Molecular testing was performed on the FNAC material, due to the young age of the patient and the suspicious cytology. In brief, DNA was directly purified from 150 *μ*l of FNAC sample (diluted in RNAprotect Cell Reagent) with QIAGEN QIAamp DNA Blood Mini Kit in accordance with manufacturer instructions. Molecular analysis was conducted by using a next-generation sequencing (NGS) commercial CE-IVD panel analysing hotspot regions of 17 cancer-related genes (*ALK*, *BRAF*, *EGFR*, *ERBB2*, *FGFR3*, *HRAS*, *IDH1*, *IDH2*, *KIT*, *KRAS*, *MET*, *NRAS*, *PDGFRA*, *PIK3CA*, *POLE*, *RET,* and *ROS1*) for single nucleotide variations and small indels (Myriapod NGS Cancer Panel DNA, Diatech Pharmacogenetics, Jesi, AN, Italy). Starting from 20 ng of DNA, libraries were prepared according to the manufacturer's instructions, diluted at 12 pM, pooled together, and sequenced on the iSeq 100 platform (Illumina Inc., San Diego, CA, USA). Data analysis was carried out on Myriapod® NGS Data Analysis Software (Diatech Pharmacogenetics); samples with at least 500x mean coverage are deemed as adequate.

**Figure 1:**
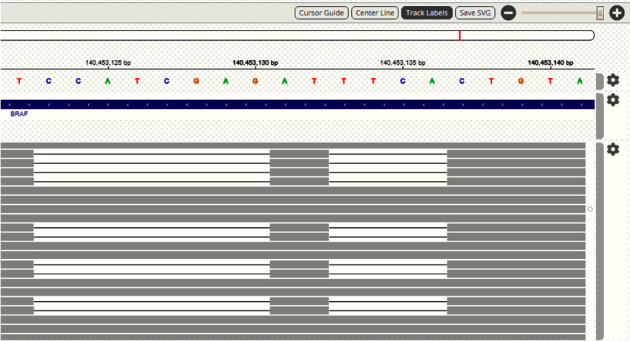
Viewing variants on the IGV (Integrative Genomics Viewer) tool.

Data analysis showed two concomitant frameshift *BRAF* variants ranging between 600 and 604 codons: c.1799_1802delTGAA, p.V600fs (variant allele frequency, VAF, 14.76%) and c.1805_1812delCTCGATGG, p.S602fs (VAF 14.84%). The Integrative Genomics Viewer (IGV) tool displayed that the variants were on the same allele (Figure 1), and actually, they resulted in an in-frame delins, namely, c.1799_1812delinsAT, p.V600_W604delinsD.

**Figure 2:**
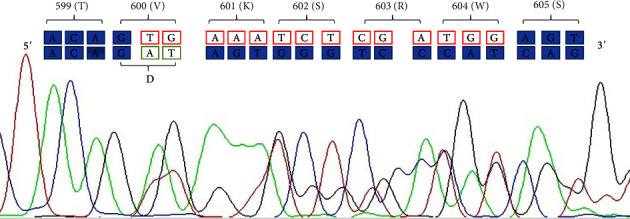
Electropherogram obtained by Sanger sequencing of *BRAF* exon 15 showing a region between codons 599 and 605 (amino acid symbol in round brackets) according to the reference sequence (NM_004333.6; transcript: ENST00000646891.2). Red codons and red-squared nucleotides are deleted and the green ones are inserted.

Because of the thyroid nodule cytology and for the presence of *BRAF* mutation, the patient underwent total thyroidectomy. The histopathological diagnosis revealed a 3 cm classic PTC infiltrating thyroid parenchyma and thyroid capsule, without microscopic extrathyroidal infiltration (pT2mNxMx); multiple foci of micro-PTC were present in the same lobe, while the contralateral lobe was normal. To conduct mutational analysis, DNA was extracted from archival formalin-fixed, paraffin-embedded tissue specimen, and PCR followed by direct Sanger sequencing was performed to analyse the sequence of BRAF exon 15, as described in previous reports [8]. The presence of the p.V600_W604delinsD mutation was confirmed on electropherogram inspection (Figure 2).

**Figure 3:**
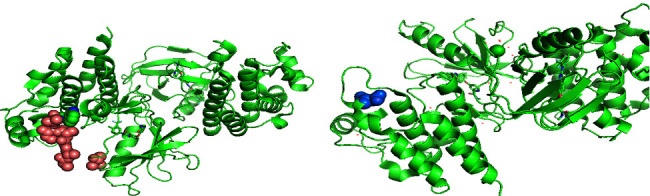
Three-dimensional protein structure of the wild-type (a) and mutant (b) BRAF protein.

To evaluate the pathogenesis of this novel mutation, we performed in silico analysis using PredictSNP (https://loschmidt.chemi.muni.cz/predictsnp/), a consensus classifier that analysed amino acid substitutions, which gives back a score predicting the possible impact on protein function employing six best-performing integrated tools (MAPP, PhD-SNP, PolyPhen-1, PolyPhen-2, SIFT, and SNAP) [11]. Moreover, the Swiss model and PyMOL software were utilized to build the three-dimensional protein structure of wild-type (Figure 3(a)) and mutant BRAF protein (Figure 3(b)). The UniProt Repository 3D protein structure model P15056 (PDB ID: 1UWH, from aa 448 to 723) Homo sapiens (Human) was used to predict the effect of mutation.

When analysed by PredictSNP, the p.V600_W604delindD mutation was predicted to be severely deleterious with an overall score of 76% (MAPP: 65%, PhD-SNP: 86%, PolyPhen-1: 74%, PolyPhen-2: 63%, SIFT: 79%, and SNAP: 85%). In addition, the modeling showed that the p.V600_W604delinsD dramatically altered the structure of the protein (Figure 3(b)).

The patient was submitted to total thyroidectomy (TT) with no complications. Pathology analysis showed a multifocal classic papillary of 3.0 cm in the right lobe (pT2(m)Nx) [12]. The specimen showed no extrathyroidal extension, angioinvasion, or lymphovascular invasion. The margins were uninvolved based on the total thyroidectomy specimen. The patient underwent a postoperative radioactive iodine ablation with 30 mCi of radioactivity and was placed on a suppressive dose of exogenous thyroid hormone (levothyroxine). On follow-up at 6 months postoperatively, the patient is found to be doing well and disease-free, with a thyroglobulin level of less than 0.1 ng/mL.

## 3. Discussion

The widespread diffusion of NGS systems has radically increased the detection rate of non-V600E *BRAF* alterations in diagnostic routine. Non-V600E *BRAF* variants have been identified in many types of cancer; with the exception of intrahepatic cholangiocarcinoma, non-V600E mutations are generally less prevalent than V600 variants [1, 13]. From recent studies, a new classification system is emerging for *BRAF* mutations based on biochemical and signalling mechanisms associated with these mutations: class I mutations promote high kinase activity, and the protein works as a monomer in a RAS-independent way (e.g., V600E); in class II mutations, proteins work as constitutively active RAS-independent dimers (e.g., K601E); and class III mutations (e.g., D594G/N/A) have low/absent kinase activity [14]. In addition, *BRAF* point mutations are considered the most common genetic variations in PTC cases; among them, p.V600E represents the most frequent detectable alterations in thyroid cancer [15–17]. Interestingly, less frequently detected *BRAF* mutations, such as p.K601E, p.K601del, and p.V599ins, have been described [18–20].

The p.K601E mutation has been classified as a RAS-like mutation being associated with follicular-patterned tumors, especially encapsulated follicular subtype PTCs [21]. This mutation seems linked with less aggressive pathologic features [8, 9].

Barollo et al. detected 5 rare *BRAF* variants using Western blot and immunofluorescence in a large group of PTCs. These alterations were previously identified in 1 FTC and in 10 PTC cases showing an overall prevalence of 1.6% (10 out of 644) of PTC and 1.6% (11/700) across thyroid cancers [22]. In a recent study, the NGS analysis of 1654 thyroid nodules showed that BRAF non-V600E mutations were present in 1.1% of nodules [9]. In this report, uncommon *BRAF* mutations were predominantly observed in follicular variants of PTCs and were linked to less aggressive behaviour, as reported in other studies [7, 23, 24]. However, in some cases, non-V600E mutations were observed in classic PTC, thus indicating that uncommon exon 15 *BRAF* mutations can be also associated with papillary architecture, as well as other *BRAF*^V600E^-like alterations. Moreover, in two cases, non-V600E mutations (i.e., p.A598_T599insILA and p.K601N) were detected in follicular thyroid carcinoma patients with distant metastases [9]. These results indicate that the spectrum of non-V600E BRAF mutations is complex, and it still needs elucidating at the molecular level and also at the clinical level.

Herein, a novel deletion/insertion c.1799_1812delinsAT (p.V600_W604delinsD) in *BRAF* exon 15 was found in a young adult patient with a classic PTC. This mutation consists of heterozygous 14 bp deletion with 2 bp insertions (AT), resulting in a complex amino acid change, with the substitution of valine 600 by aspartate and the deletion of lysine 601, serine 602, arginine 603, and tryptophan 604. To our knowledge, this genetic event has never been reported before in PTC, and further studies are needed to elucidate its functional consequences on *BRAF* activation status.

Mutant *BRAF* has been implicated in the pathogenesis of different types of cancer, but the clinical significance of mutations outside codon 600 is largely unknown [25]. Expanding the knowledge of non-V600E *BRAF* mutations represents an open challenge with potential implications for patients' management and treatment.

## Data Availability

The data used to support the findings of this study are included within the article.
